# Non-Invasive Diagnosis of Diabetes by Volatile Organic Compounds in Urine Using FAIMS and Fox4000 Electronic Nose

**DOI:** 10.3390/bios8040121

**Published:** 2018-12-01

**Authors:** Siavash Esfahani, Alfian Wicaksono, Ella Mozdiak, Ramesh P. Arasaradnam, James A. Covington

**Affiliations:** 1 School of Engineering, University of Warwick, Coventry CV4 7AL, UK; a.wicaksono@warwick.ac.uk (A.W.); J.A.Covington@warwick.ac.uk (J.A.C.); 2 Department of Gastroenterology, University Hospital Coventry and Warwickshire, Coventry, CV2 2DX, UK; ella.mozdiak@nhs.net (E.M.); R.Arasaradnam@warwick.ac.uk (R.P.A.); 3 School of Applied Biological Sciences, University of Coventry, Coventry CV1 5FB, UK; 4 Warwick Medical School, University of Warwick, Coventry CV4 7AL, UK

**Keywords:** electronic nose, biosensor, diabetes, FOX 4000, FAIMS, urine sample, non-invasive diagnosis, medical application, volatile organic compounds (VOCs)

## Abstract

The electronic nose (eNose) is an instrument designed to mimic the human olfactory system. Usage of eNose in medical applications is more popular than ever, due to its low costs and non-invasive nature. The eNose sniffs the gases and vapours that emanate from human waste (urine, breath, and stool) for the diagnosis of variety of diseases. Diabetes mellitus type 2 (DM2) affects 8.3% of adults in the world, with 43% being underdiagnosed, resulting in 4.9 million deaths per year. In this study, we investigated the potential of urinary volatile organic compounds (VOCs) as novel non-invasive diagnostic biomarker for diabetes. In addition, we investigated the influence of sample age on the diagnostic accuracy of urinary VOCs. We analysed 140 urine samples (73 DM2, 67 healthy) with Field-Asymmetric Ion Mobility Spectrometry (FAIMS); a type of eNose; and FOX 4000 (AlphaM.O.S, Toulouse, France). Urine samples were collected at UHCW NHS Trust clinics over 4 years and stored at −80 °C within two hours of collection. Four different classifiers were used for classification, specifically Sparse Logistic Regression, Random Forest, Gaussian Process, and Support Vector on both FAIMS and FOX4000. Both eNoses showed their capability of diagnosing DM2 from controls and the effect of sample age on the discrimination. FAIMS samples were analysed for all samples aged 0–4 years (AUC: 88%, sensitivity: 87%, specificity: 82%) and then sub group samples aged less than a year (AUC (Area Under the Curve): 94%, Sensitivity: 92%, specificity: 100%). FOX4000 samples were analysed for all samples aged 0–4 years (AUC: 85%, sensitivity: 77%, specificity: 85%) and a sub group samples aged less than 18 months: (AUC: 94%, sensitivity: 90%, specificity: 89%). We demonstrated that FAIMS and FOX 4000 eNoses can discriminate DM2 from controls using urinary VOCs. In addition, we showed that urine sample age affects discriminative accuracy.

## 1. Introduction

The growing rate of diabetes and its related diseases is becoming a worldwide major health concern. The motivation of this paper was to make use of a technology called the “electronic nose” (eNose) for diagnosing diabetes. Using eNose technology with urinary volatile organic compounds (VOCs) is attractive as it allows non-invasive monitoring of various molecular constituents in urine. Trace gases in urine are linked to metabolic reactions and diseases.

The mimicry of a biological olfactory system, called the electronic nose, was developed in the early 1980s [1]. The electronic nose contains arrays of sensors that analyses the sample as a whole complex mixture, not identifying a specific chemical [2,3]. By developing technology and increasing demand for non-invasive methods for diagnosis diseases, the electronic nose is becoming a promising instrument in the medical domain. Commercial and experimental electronic noses have been developed for diagnosis of a wide range of diseases such as lung cancer [4], breast cancer [5], brain cancer [6] and melanoma [7], prostate cancer [8], colorectal cancer [2], asthma [9], and many other diseases. There are only few studies on diagnosing diabetes using urinary VOCs with eNose instruments [10,11].

Currently, one of the urgent public medical issues is the fast-growing number of people with diabetes. According to statistics, the number of people worldwide with diabetes in 2017 was estimated to be 425 million, with 1 of 2 adults remaining undiagnosed [12]. In the UK, the number of people diagnosed with type 2 diabetes was just under 3.7 million people in 2017, with a further estimated 1 million people remaining undiagnosed, which is better than worldwide figures [13]. It is a major health concern especially in the under 20 s, where the numbers of diabetic children are rapidly increasing. In the UK alone, around 31,500 patients under the age of 19 have diabetes [12]. According to the National Diabetes Audit (NDA) report, 24,000 patients suffering from diabetes have early death each year (65 patients a day) [14]. From a financial cost point of view, 10% of the NHS budget is spent on diabetes.

Our approach was to undertake a pilot study to investigate if urinary VOCs (volatile organic compounds) could be used as a non-invasive means to identify patients with type 2 diabetes Mellitus (T2DM). These samples were collected over a four-and-a-half-year period and stored at −80 °C and then analysed using by Owlstone Lonestar FAIMS and FOX4000, as two types of electronic nose. From our previous study, it was discovered that samples over 12 months old will not emit sufficient VOCs for diagnostic purposes [15], hence this paper will focus more on analysing samples less than 12 months old for diagnosing diabetes samples compared to healthy control.

## 2. Materials and Methods

### 2.1. Sample Preparation

One hundred and thirty-eight patients were recruited at the University Hospital Coventry & Warwickshire, UK. Each recruit provided a urine sample, which was collected in a clinic and frozen at −80 °C within two hours over a four-and-a-half-year period. Seventy-one samples came from patients with type 2 diabetes, with a further 67 samples from healthy controls. Scientific and ethical approval was obtained from the Warwickshire Research & Development Department and Warwickshire Ethics Committee 09/H1211/38. Written informed consent was obtained from all patients who participated in the study. For analysis, samples were thawed to 4 °C in a laboratory fridge for 24 h prior to testing to minimise chemical loss. Demographic details of patients are shown in Table 1.

### 2.2. FAIMS Chemical Analyser

**Figure 1:**
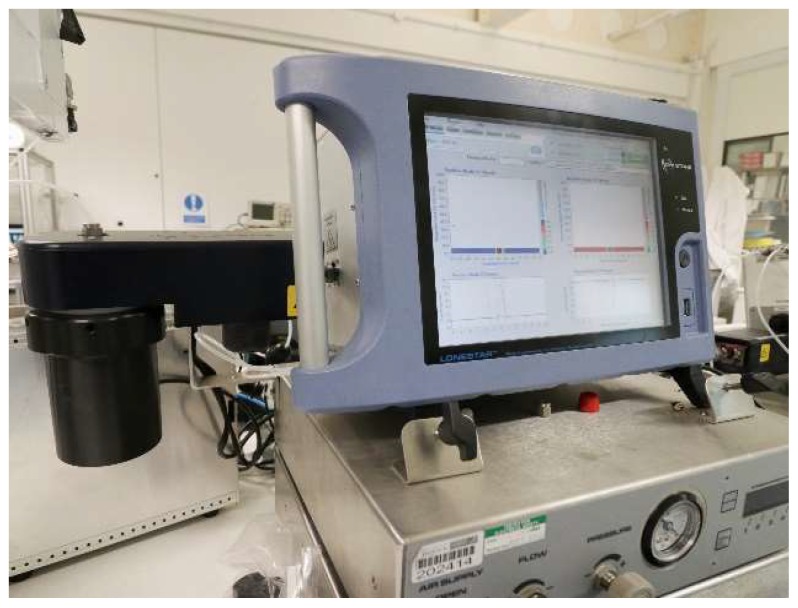
Field-asymmetric ion mobility spectrometer (FAIMS) setup.

**Figure 2:**
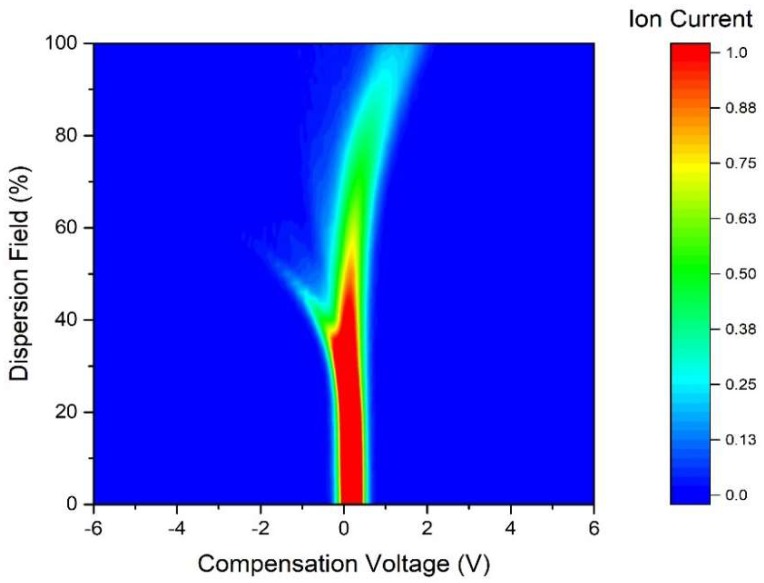
Typical FAIMS output responding to urine vapour.

A commercial FAIMS (Field-Asymmetric Ion Mobility Spectrometer) device was used in this study, specifically a Lonestar instrument (Owlstone, Cambridge, UK). It is able to separate complex chemical mixtures by measuring the difference in mobility of ionised molecules in high electric fields, thus it measures a physical property of a gas or vapour. The Lonestar was setup to use dynamic headspace sampling, using an ATLAS sampling system (Owlstone, Cambridge, UK), which controls of the flow rate and the temperature of the sample. The unit pushes clean/dry air over the surface of the urine and into the Lonestar instrument. The chemical components are then ionised (Ni-63 source) and pushed through two parallel plates. An asynchronous waveform is applied to these plates, consisting of a high electric field for a short period of time, followed by an inverse potential of low electric field, but with the time x electric field being equal. If a molecule’s mobility is constant with electric field, the ion exits the plates and is detected. However, if the electric field attracts or repels an ion, it drifts towards a plate and loses its change when it makes contact. To remove this drift, a constant voltage (called the compensation voltage) is applied, thus by scanning through different compensation voltages, we can measure a range of mobilities. Both the magnitude of the electric field (called the dispersion field) and the compensation voltage is scanned to create a 3D map of molecular mobilities [16]. Figure 1 shows the FAIMS instrument setup and Figure 2 shows the typical output of FAIMS instrument. In this experiment, 5 mL of urine were aliquoted from each sample into a 10 mL glass vial and placed into an ATLAS sample system and followed a similar setup to one previously used by our group [17,18]. This heated the sample to 40 ± 0.1 °C. Each sample was tested three times sequentially, with each run having a flow rate over the sample of 200 mL/min of clean dry air. Further make-up air was added to create a total flow rate of 2 L/min. The FAIMS was scanned from 0% to 99% dispersion field in 51 steps, −6 V to +6 V compensation voltage in 512 steps and both positive and negative ions were detected to create a test file composed of 52,224 data points.

### 2.3. Electronic Nose

A commercial electronic nose (FOX 4000 with HS100 autosampler, Alpha M.O.S, Toulouse, France) was used in this study. The Fox 4000 consists of an injection system, sensor chambers, mass flow controller, and acquisition board with microcontroller. The electronic nose contains 18 metal oxide gas sensors that are placed in three chambers and were calibrated regularly in line with the manufacturer’s recommended procedures to ensure stability. These three chambers are called T, P, and LY. All the sensors’ names and their application are indicated in Table 2.

The basic operation principle of the electronic nose is based on the sensors’ electronic resistance changes in response to the presence of volatile compounds. In our case, the output response was calculated by the formula in Equation (1) [19].

(1) $$R=\left(R_{0}-R_{T}\right)/R_{0}$$

where *R* is response of sensor, *R*_0_ is initial resistance of metal oxide sensor at time 0, and *R_T_* is sensor’s conductance value.

**Figure 3:**
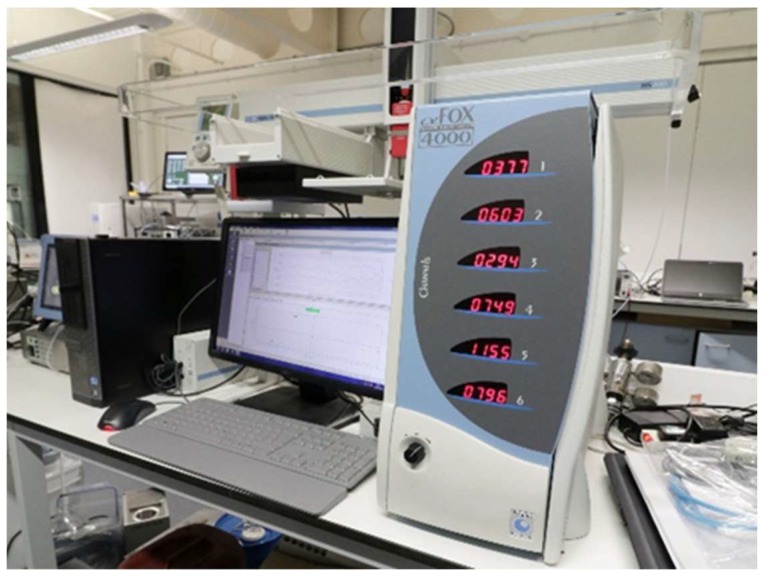
FOX 4000 setup.

**Figure 4:**
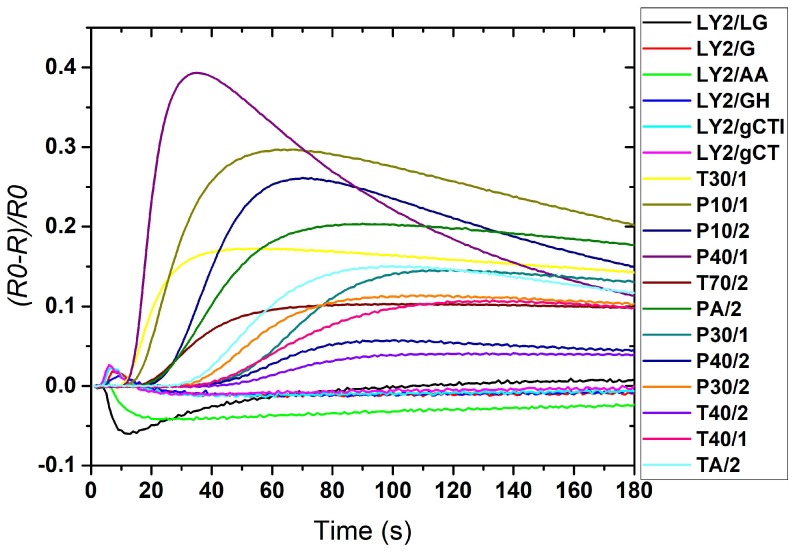
Typical response of a FOX 4000 to a urine sample.

Figure 3 shows the setup of the FOX 4000 instrument. Figure 4 shows a typical response of a FOX 4000 with 18 sensors to a diabetic urine sample’s volatile compounds. Each curve signifies one sensor’s response. The concentration and nature of the sensed molecules plus the type of metal oxide sensors used in the eNose are the three main reasons for the size of the sensor’s response [9].

The samples were agitated and heated to 40 °C for 10 min before 2.5 ml of the sample headspace was injected into the electronic nose (flow rate over the sensors was 200 ml/min of zero air, data was recorded for 180 s at a sample rate of 1 Hz). The data from the FOX4000 are generated by sampling each of the 18 metal oxide sensors at 180 data points over 180 s. These readings are concatenated into a single vector representing a single sample, and thus the raw data are of dimension 320.

## 3. Results

### 3.1. FAIMS Analysis

**Figure 5:**
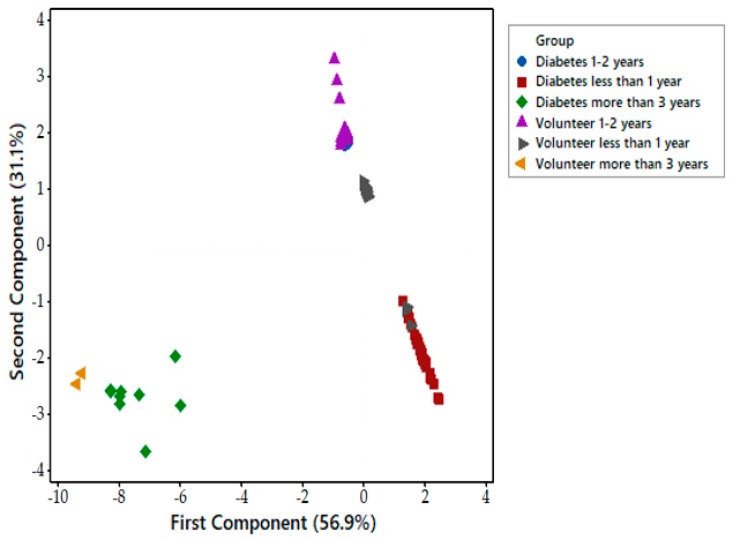
Principal component analysis (PCA) of samples between 0 and 4 years.

**Figure 6:**
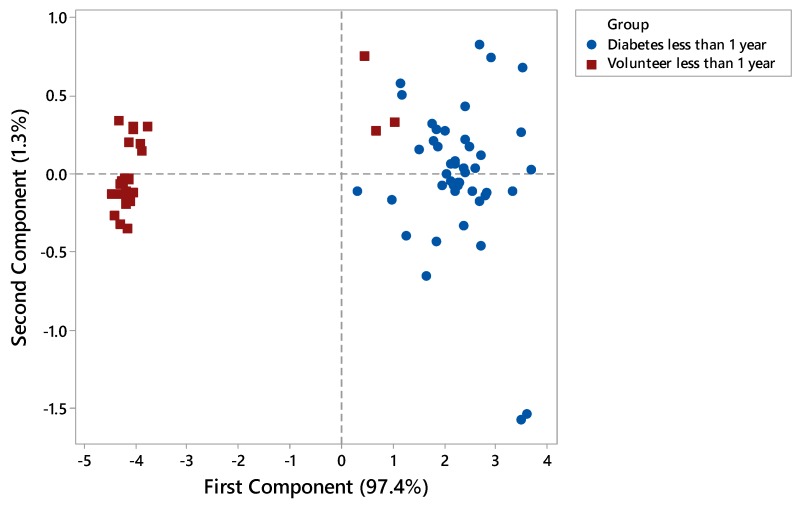
PCA analysis of samples less than 1 year old.

Each FAIMS dataset consisted of 52,224 data points that are stored in a 512 × 102 matrix. The first step of data processing was performing a pre-processing step by applying 2D wavelet transform (using Daubechies D4 wavelets) to each data set. This step aimed to decompose the signal and extract subtle chemical signals within a wider range of the signal. The 2D wavelet transform will concentrate the chemical information into several levels, which consist of a small number of wavelet coefficients. These coefficients would then be the input. We randomly divided the input into two sets, with 70% used as a training/validation set, and 30% as a test set. Ten-fold cross-validation was applied to the training and validation set in which, within each fold, supervised features selection was performed using Wilcoxon rank sum test by calculating the *p*-values for every pair of features in the training set. Principal component analysis (PCA) was performed to see distribution of data in the scatter plot. The ten most statistically important features, which had the lowest *p*-values, were then used to train the classifier algorithms. Four different classifiers were used for prediction, specifically Sparse Logistic Regression, Random Forest, Gaussian Process, and Support Vector Machines. The hyperparameter of the classifier was then tuned by comparing the error function using an independent validation set. This step was used to minimise overfitting. Finally, the performance of classifier algorithms and their diagnostic capabilities were calculated using an independent test set and were displayed in a graphical plot called Receiver Operator Characteristic (ROC) curve. The ROC provides information about Area Under Curve (AUC), sensitivity, specificity, Positive Predictive Value (PPV), Negative Predictive Value (NPV), and *p*-values. Sample age affected the vapour emission from the sample. Figure 5 shows that using the PCA method on the whole group of samples from zero to four years is not sufficient to distinguish diabetic samples from control ones. However, by separating samples by age and applying the PCA method, the results showed better separation between diabetes and control groups of newer samples. Figure 6 is related to samples of age less than 1 year, as expected, where the separation is clear between two different groups. Only three samples have cross selectivity between diabetes and control groups.

**Figure 7:**
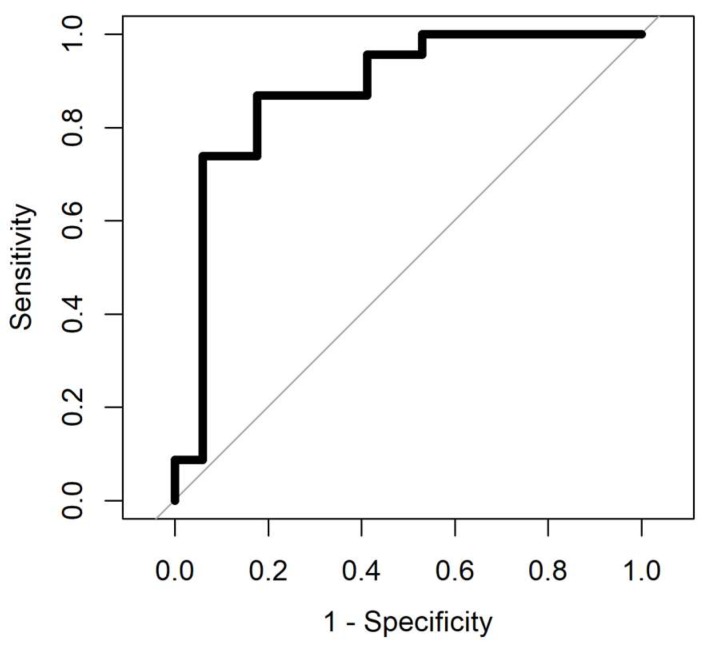
Receiver operating characteristic (ROC) analysis of samples aged 0–4 years. Gaussian process (AUC = 0.88).

**Figure 8:**
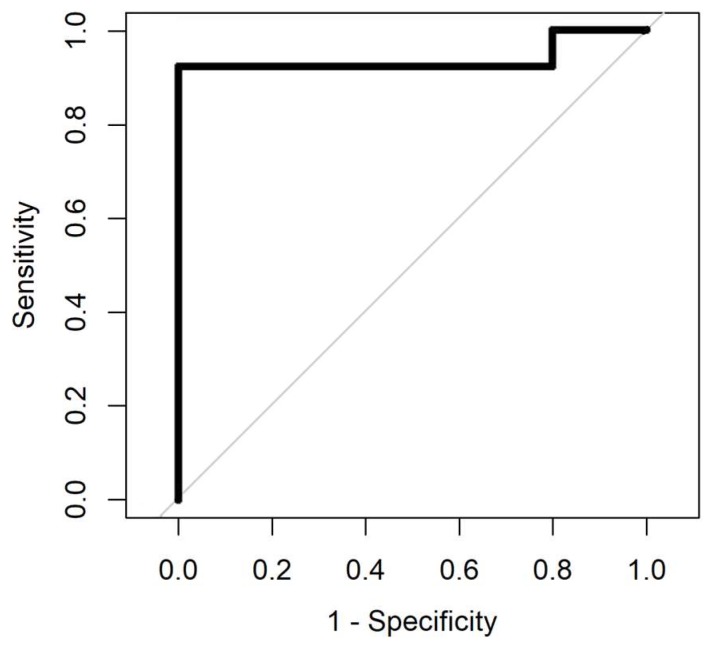
Receiver operating characteristic (ROC) analysis of samples aged less than a year. Gaussian Process (AUC = 0.94).

The result of ROC analysis with four different methods for samples aged 0–4 years and samples aged less than a year are summarised in Table 3 and Table 4. The ROC analysis was processed for samples with four different analysis methods. Gaussian processing appears to be the best method since it has the highest area under curve and from a medical perspective, it is important to have low negative predictive value (NPV), which this method has compared to the others. The area under the curve in the ROC shows how good the separation is. The max value for area under the curve in the ROC is 1. AUC values for all methods, for data with less than a year, is more than 0.9, and it is below 0.9 for samples aged 0–4 years. Figure 7 and Figure 8 show the ROC analysis of the two groups of sample age. As is clear from the figures, better classification performance is achieved for samples with less storage time.

### 3.2. Electronic Nose Analysis

**Figure 9:**
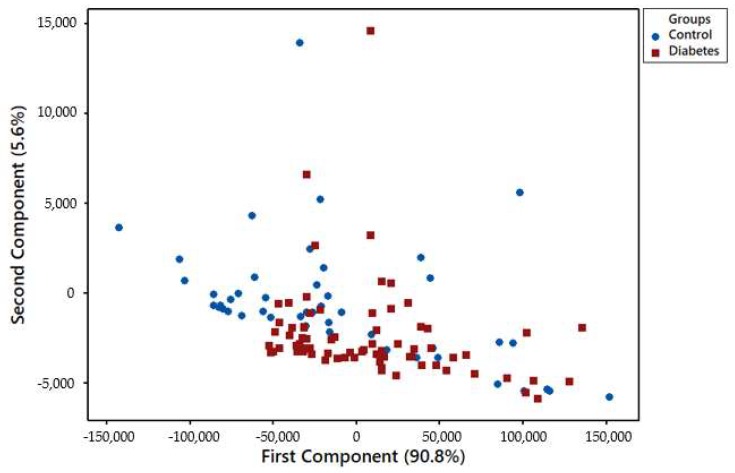
PCA components 1 and 2 show 4-year-old disease and control samples separation.

**Figure 10:**
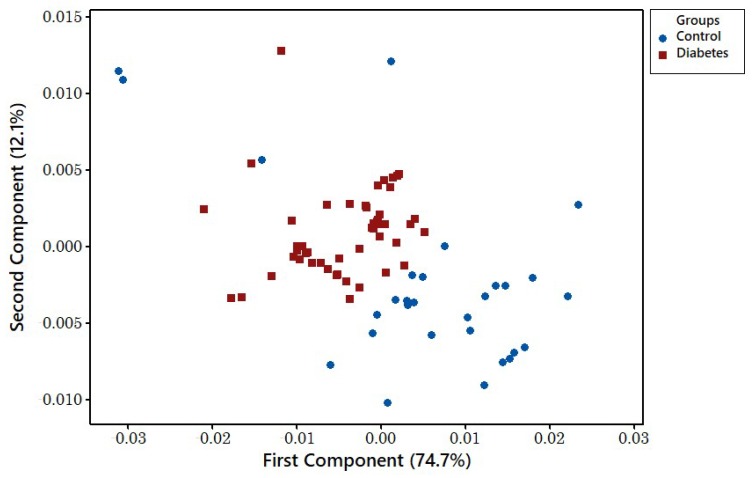
PCA components 1 and 2 show 18-month-old disease and control samples separation.

PCA analysis was used for classification of the features that were extracted by dividing the maximum resistance by the baseline resistance. Plotting only the first and second PCA components shows the disease classification has been affected by the urine sample’s storage age. Figure 9 illustrates the diabetes and control samples collected 4 years prior to analysis, for which the classification is not performed appropriately. Figure 10 shows the classification for diabetes and control samples collected and tested in less than 18 months. The results show that newer samples are tightly clustered and sufficiently separated from the disease class in comparison to the group of older samples.

**Figure 11:**
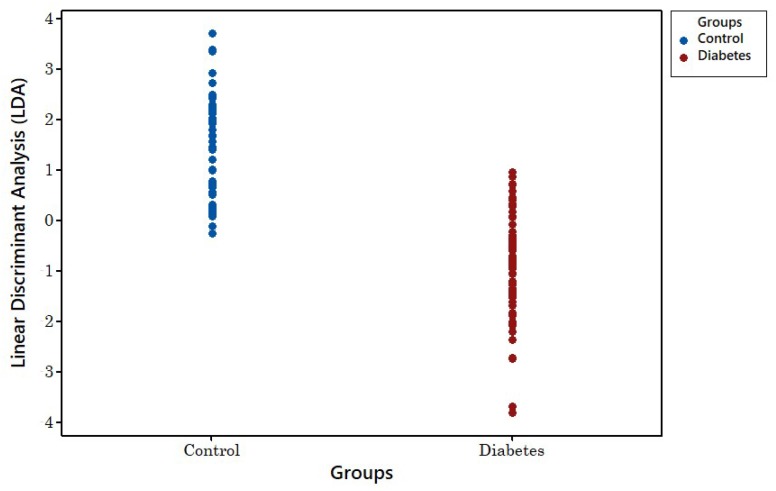
LDA classification of 4-year-old disease and control samples.

**Figure 12:**
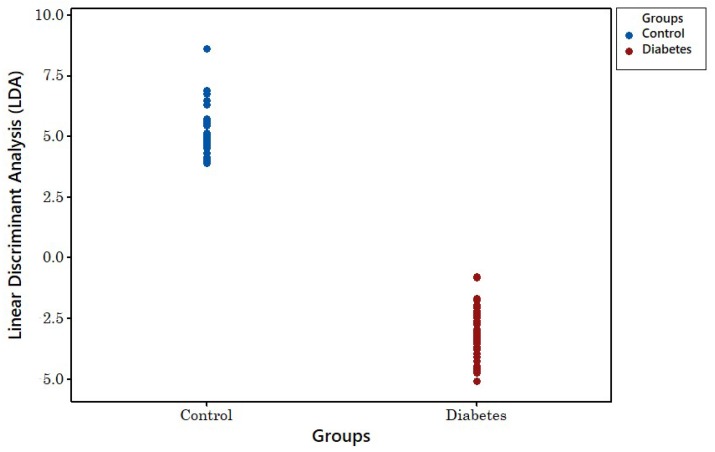
LDA classification of 18-month-old disease and control samples.

Figure 11 shows the linear discrimination analysis (LDA) result for the four-year-old samples. As can be seen, there is no clear separation between groups. Figure 12 shows the LDA method’s result for newer samples with less than 18 months of age. This shows clear separation between both groups.

**Figure 13:**
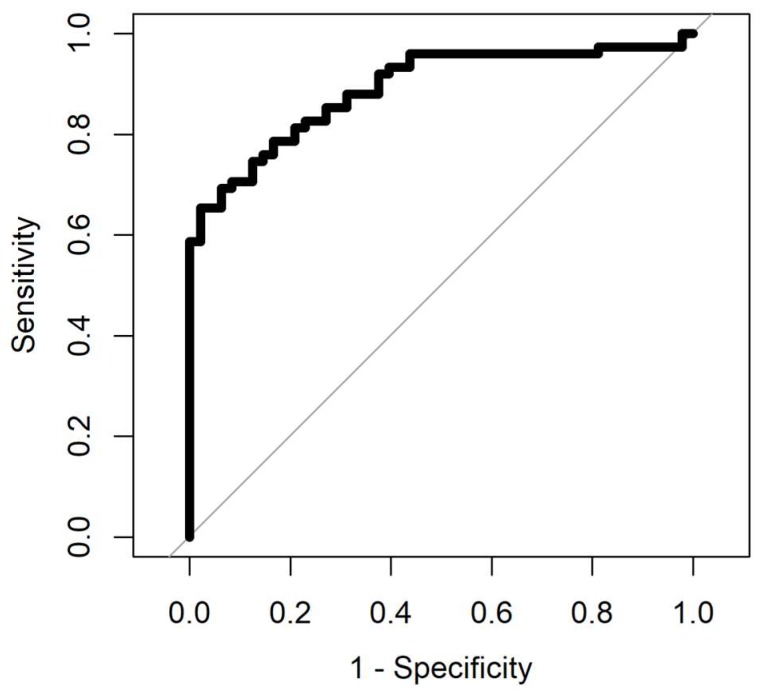
ROC with Boruta package analysis for data from samples up to 4 years old. Sparse Logistic Regression (AUC = 0.89).

**Figure 14:**
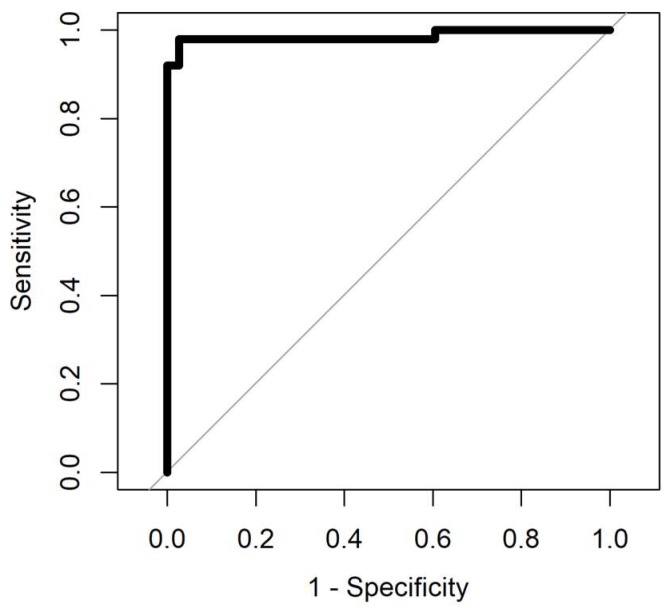
ROC with Boruta package analysis for data from samples less than 18 months old. Sparse Logistic Regression (AUC = 0.99).

To analyse this set of data, maximum variances of sensor resistance were chosen as features. Then, the four classifiers were analysed using the Boruta package [20]. Four different methods were used to ensure validity of the results. Table 5 summarises the results of these methods for 4-year-old samples and Table 6 summarises the result for samples less than 18 months old. From Table 5 and Table 6, it is clear that Sparse Logistic Regression worked more efficiently than the other methods since it has a greater area under curve value. Figure 13 and Figure 14 indicate the ROC analysis of 128 samples of VOCs differentiating between diabetes and control samples for two different sample age groups.

## 4. Discussion

This paper has introduced diabetes as a major global health concern, affecting 1 in 12 of the population. The power of FAIMS and the FOX 4000 eNose to distinguish healthy and diabetic patients is considerable. Using an electronic nose, along with statistical and machine learning techniques, it was shown that we can accurately classify diabetic patients from healthy controls using only the aromas emanating from a urine sample. High prediction accuracy was achieved by combining PCA with a sparse logistic regression and a Gaussian process classifier. No single sensor was found to be able to distinguish healthy and disease patients, yet combining all sensors allows a high degree of predictive accuracy. It offers hope in developing a low-cost, point of care, rapid diagnostic tool that could potentially be an alternative non-invasive means to diagnose and, in the future, monitor the progression of diabetes.

The secondary result of this paper is proof that the vapours emanating from a stored urine sample are affected by storage time, as demonstrated by using the FOX 4000 and FAIMS electronic nose instruments. In most cases, samples would be tested well before this four-and-a-half-year period. Our study suggests that this is not feasible when dealing with urine samples for gas analysis. The results presented in this paper suggest that the optimal timing for urine analysis is less than 12 months and certainly not beyond this sample age.

## 5. Conclusions

Diabetes affects a large proportion of the world population and results in millions of deaths every year. Currently more than 40% of individuals with type 2 diabetes are undiagnosed. Here, an Alpha M.O.S FOX-4000 and FAIMS electronic nose were used to analyse urinary aromas from subjects with type 2 diabetes and healthy controls. By performing PCA and applying a classification algorithm, a high predictive accuracy was achieved. This study provides evidence suggesting that it may be possible to use urinary gas phase bio-markers to diagnose and monitor diabetes. Discriminating diabetic from control samples with above 95% accuracy proves that it is possible to diagnose diabetes from VOCs emitted from urine sample with eNose instruments. FAIMS can distinguish between diabetes and control samples that are less than a year old with sensitivity greater than 90% and specificity greater than 80%. FOX 4000 can separate diabetic from control samples with sensitivity and specificity above 90%. Also, it is suggested that samples under 12 months of age produce enough VOCs for urine analysis using an eNose.

## Figures and Tables

**Table 1:** Demographic information of used urinary samples (incomplete data for 2 diabetic patients).

Demographic Data	Diabetes	Control
Male (%)	27 (39.1)	43 (64.2)
Female (%)	42 (60.9)	24 (35.8)
Median age (year)	57	53.5
Mean alcohol (units/week)	1.8	1.09
Median BMI	39.7	26.1

**Table 2:** α-FOX4000 eNose sensor arrays and their applications.

Sensor No.	References	Description
S1	LY2/LG	Oxidising gas
S2	LY2/G	Ammonia, carbon monoxide
S3	LY2/AA	Ethanol
S4	LY2/GH	Ammonia/ Organic amines
S5	LY2/gCTL	Hydrogen sulfide
S6	LY2/gCT	Propane/Butane
S7	T30/1	Organic solvents
S8	P10/1	Hydrocarbons
S9	P10/2	Methane
S10	P40/1	Fluorine
S11	T70/2	Aromatic compounds
S12	PA/2	Ethanol, Ammonia/Organic amines
S13	P30/1	Polar compounds (Ethanol)
S14	P40/2	Heteroatom/Chloride/Aldehydes
S15	P30/2	Alcohol
S16	T40/2	Aldehydes
S17	T40/1	Chlorinated compounds
S18	TA/2	Air quality

**Table 3:** Summary of the ROC (Receiver Operator Characteristic) analysis details for samples 0–4 years old.

Methods	AUC	Sensitivity	Specificity	PPV	NPV	*p*-Value
**Sparse Logistic Regression**	0.89 (0.79–0.99)	0.74 (0.51–0.9)	0.88 (0.63–0.99)	0.89	0.71	4.368 × 10^−6^
**Random Forest**	0.86 (0.74–0.98)	0.78 (0.56–0.92)	0.82 (0.56–0.96)	0.86	0.74	6.690 × 10^−5^
**Gaussian Process**	0.88 (0.76–1)	0.87 (0.66–0.97)	0.82 (0.56–0.96)	0.87	0.82	7.187 × 10^−6^
**Support Vector Machine**	0.88 (0.77–0.99)	0.74 (0.51–0.9)	0.94 (0.71–0.99)	0.94	0.73	7.189 × 10^−6^

**Table 4:** Summary of the ROC analysis details for samples less than 1 year old.

Methods	AUC	Sensitivity	Specificity	PPV	NPV	*p*-Value
**Sparse Logistic Regression**	0.9 (0.7–1)	1 (0.75–1)	0.9 (0.55–0.99)	0.93	1	3.199 × 10^−4^
**Random Forest**	0.93 (0.79–1)	1 (0.75–1)	0.9 (0.55–0.98)	0.93	1	1.419 × 10^−4^
**Gaussian Process**	0.94 (0.82–1)	0.92 (0.64–1)	1 (0.69–1)	1	0.91	5.856 × 10^−5^
**Support Vector Machine**	0.9 (0.7–1)	1 (0.75–1)	0.9 (0.55–0.99)	0.93	1	3.199 × 10^−4^

**Table 5:** ROC with Boruta package analysis of data from 4-year-old samples.

Methods	AUC	Sensitivity	Specificity	PPV	NPV	*p*-Value
**Sparse Logistic Regression**	0.89 (0.83–0.95)	0.65 (0.53–0.76)	0.98 (0.89–1)	0.98	0.64	1.583 × 10^−13^
**Random Forest**	0.89 (0.84–0.95)	0.69 (0.58–0.79)	0.9 (0.77–0.97)	0.91	0.65	1.088 × 10^−13^
**Gaussian Process**	0.85 (0.78–0.92)	0.77 (0.66–0.86)	0.85 (0.72–0.94)	0.89	0.71	4.04 × 10^−11^
**Support Vector Machine**	0.78 (0.69–0.88)	0.88 (0.78–0.94)	0.69 (0.54–0.81)	0.81	0.79	8.529 × 10^−8^

**Table 6:** ROC with Boruta package analysis for data from 18-month-old samples.

Methods	AUC	Sensitivity	Specificity	PPV	NPV	*p*-Value
**Sparse Logistic Regression**	0.99 (0.96–1)	0.98 (0.89–1)	0.97 (0.86–1)	0.98	0.97	3.639 × 10^−15^
**Random Forest**	0.97 (0.94–1)	0.98 (0.89–1)	0.87 (0.72–0.96)	0.91	0.97	4.317 × 10^−14^
**Gaussian Process**	0.94 (0.89–0.99)	0.9 (0.78–0.97)	0.89 (0.75–0.97)	0.92	0.87	9.162 × 10^−13^
**Support Vector Machine**	0.94 (0.87–1)	0.98 (0.89–1)	0.89 (0.75–0.97)	0.92	0.97	9.733 × 10^−13^
